# Deciphering the structure of a multi-drug resistant *Acinetobacter baumannii* short-chain dehydrogenase reductase

**DOI:** 10.1371/journal.pone.0297751

**Published:** 2024-02-23

**Authors:** Mahdi Abedinzadeh Shahri, Paniz Shirmast, Seyed Mohammad Ghafoori, Jade Kenneth Forwood

**Affiliations:** 1 School of Dentistry and Medical Sciences, Charles Sturt University, Wagga Wagga, NSW, Australia; 2 Menzies Health Institute Queensland and School of Medical Sciences, Griffith University, Gold Coast, QLD, Australia; Rani Lakshmi Bai Central Agricultural University, INDIA

## Abstract

The rapidly increasing threat of multi-drug-resistant Acinetobacter baumannii infections globally, encompassing a range of clinical manifestations from skin and soft tissue infections to life-threatening conditions like meningitis and pneumonia, underscores an urgent need for novel therapeutic strategies. These infections, prevalent in both hospital and community settings, present a formidable challenge to the healthcare system due to the bacterium’s widespread nature and dwindling effective treatment options. Against this backdrop, the exploration of bacterial short-chain dehydrogenase reductases (SDRs) emerges as a promising avenue. These enzymes play pivotal roles in various critical bacterial processes, including fatty acid synthesis, homeostasis, metabolism, and contributing to drug resistance mechanisms. In this study, we present the first examination of the X-ray crystallographic structure of an uncharacterized SDR enzyme from *A*. *baumannii*. The tertiary structure of this SDR is distinguished by a central parallel β-sheet, consisting of seven strands, which is flanked by eight α-helices. This configuration exhibits structural parallels with other enzymes in the SDR family, underscoring a conserved architectural theme within this enzyme class. Despite the current ambiguity regarding the enzyme’s natural substrate, the importance of many SDR enzymes as targets in anti-bacterial agent design is well-established. Therefore, the detailed structural insights provided in this study open new pathways for the in-silico design of therapeutic agents. By offering a structural blueprint, our findings may provide a platform for future research aimed at developing targeted treatments against this and other multi-drug-resistant infections.

## Introduction

*Acinetobacter baumannii* is an opportunistic Gram-negative pathogen and major cause of nosocomial infections worldwide [1,2]. The Acinetobacter genus comprise more than 50 species to date [3], with members such as *A*. *baumannii*, *A*. *pittii*, *A*. *nosocomialis*, *A*. *seifertii and A*. *dijkshoorniae* causing human disease [4]. *A*. *baumannii*, the most serious cause of problematic nosocomial infections, is an “ESKAPE” pathogen (which include *Enterococcus faecium*, *Staphylococcus aureus*, *Klebsiella pneumoniae*, *Acinetobacter baumannii*, *Pseudomonas aeruginosa*, and *Enterobacter* species [5]), and carries antimicrobial resistance genes that make treatment options challenging [6]. In recent years concerns have been raised due to the significant morbidity and mortality of patients infected with multi-drug-resistant *A*. *baumannii*, and the high prevalence of this pathogen especially in hospital settings [7–9]. To date, *A*. *baumannii*, an invasive pathogen, has been reported to be responsible for hospital-acquired infections, especially in intensive care unit (ICU) patients [10,11]. Antibiotic resistance is a major challenge among *A*. *baumannii* strains, and has been categorized as a global threat by the World Health Organization and Centers for Disease Control and Prevention [12,13]. In addition to antibiotic resistance, *A*. *baumannii* with its highly adaptive nature, utilizes different virulence factors that allows the bacteria to make biofilms, adhere to surfaces, escape host immunity, and survive in the environment [14,15]. Moreover, intrinsic antibiotic resistance and acquired resistance via mutations and horizontal gene transfer in *A*. *baumannii* are responsible for developing multidrug resistance to beta-lactams, aminoglycosides, fluoroquinolones, tetracyclines and tigecycline, macrolides, lincosamides, and chloramphenicol [16,17]. Aminoglycosides, which are effective against many gram-negative infections, are largely unsuccessful in treating *A*. *baumannii* infections [18]. Moreover, aminoglycoside modifying enzymes (AMEs), including phosphotransferases, adenyltransferases, and acetyltransferases are considered to be a significant cause of aminoglycosides resistance in *A*. *baumannii* [19].

Short-chain dehydrogenases/reductases (SDR) play a variety of roles in metabolism, including those related to fatty acids, sugar, and hormone ligand levels, transcriptional control, and apoptosis [20]. A wide range of essential biological processes in prokaryotes are also carried out by members of SDRs such as fatty acid synthesis, homeostasis, lipid metabolic process, intermediate metabolism, and bacterial drug resistance [21–23]. Substrates for SDR enzymes vary considerably ranging from xenobiotics as well as alcohols, sugars, steroids, and aromatic molecules, and often these enzymes share little sequence similarity. However, their structure is often highly conserved, and harbour a classic Rossman fold domain with a core β-sheet, flanked by α-helices. They contain tyrosine, lysine, serine, and/or asparagine residues at the active site [24,25], where tyrosine serves as the catalytic base, serine assists with substrate stabilization, and lysine interacts with the nicotinamide ribose of the cofactor NAD(P) [26]. The N-terminal region of SDRs are generally more highly conserved than the C-terminal region, a reflection that co-factor binding occurs within the N-terminal domain, while the substrate binding occurs within the C-terminal domain [27]. SDRs are considered as a crucial factor in *E*. *coli*, *P*. *aeruginosa*, *S*. *aureus*, *S*. *typhimurium*, *M*. *tuberculosis*, *and in A*. *baumannii* [28]. As the only enzyme able to carry out the reduction of β-ketoacyl ACP into β-hydroacyl ACP thioesters, FabG enzymes are desirable targets for the development of novel inhibitors against gram negative bacteria, including *A*. *baumannii* [29]. Moreover, many SDR enzymes have shown potential as pharmaceutical targets for hormone-related and metabolic diseases including obesity and diabetes, and infectious diseases [30,31]. Due to the importance of SDR enzymes in prokaryote function, and the urgent and critical need to develop new antibacterial agents, in this study we present the structure of an SDR enzyme from *A*. *baumannii* that may be used as a basis for *in silico* design and testing of therapeutic agents. Our study is significant as it unveils the first-ever structure of a previously uncharacterized SDR from *A*. *baumannii*. The delineation the enzyme’s structure may be useful in docking both potential substrates as well as inhibitors for in silico drug development. Should this SDR enzyme prove vital in *A*. *baumannii’s* function or antibiotic resistance, our structural work will provide an important platform for potential therapeutic strategies that could tackle antibiotic resistance in *A*. *baumannii* infections.

## Material and methods

### Cloning and protein expression

The *A*. *baumannii* SDR nucleotide sequence (UniProt accession number: A0A0D5YL95) was cloned into the expression vector pMCSG21 at the SspI site [32]. The full protein sequence, including the His tag (green) and TEV cleavage site (red) is:

MHHHHHHSSGVDLGTENLYFQ/SNAMKLDLQNKIAVVSGSTSGIGLGIAKGLASAGATVVVVGRKQAGVDEAIAHIRQSVPEASLRGVDADLTTEQGAAALFAAEPKADILVNNLGIFNDEDFFSVPDEEWMRFYQVNVLSGVRLARHYAPSMVEQGWGRIIFISSESGVAIPGDMINYGVTKSANLAVSHGLAKRLAGTGVTVNAVLPGPTFTDGLENMLADAAAKAGRSTRDQADEFVKVLRPSSIIQRAAEVDEVANMVVYIASPLSSATSGAALRVDGGVVDTLV. To produce recombinant protein, the expression vector harbouring an N-terminal HIS affinity tag and (Tobacco Etch Virus) TEV cleavage site was transformed into competent *E*. *coli* BL21(DE3) pLysS cells. A starter culture of 5 mL of LB broth was incubated at 37°C overnight, from which 100 μL was added to 1 L of auto-induction media, and incubated at room temperature for 36 h. Cells were harvested by centrifugation and resuspended in ‘Buffer A’ containing 20 mM imidazole, 300 mM NaCl, 50 mM phosphate buffer pH 8.0, and stored at -20°C until protein purification.

### Protein purification and crystallization

The SDR enzyme was purified by lysing the bacterial cell membrane via two freeze–thaw cycles and addition of 2 mg/mL lysozyme and 0.025 mg/mL DNAse. The lysate was clarified via centrifugation and passed over a 5 mL Nickel-Sepharose HisTrap HP column (GE Healthcare). The column was washed with 10-column volumes of Buffer A to remove unbound contaminants, and the SDR enzyme eluted using a gradient of elution ’Buffer B’ (50 mM phosphate buffer pH 8.0, 300 mM NaCl, and 500 mM imidazole) over 5-column volumes. The eluate was treated with TEV protease to remove the affinity tag, then further purified using a Superdex 200 26/60 column (GE Healthcare) in tris-buffered saline (50 mM Tris pH 8.0, 125 mM NaCl). The homogenous peak from gel filtration was collected and concentrated using a 10 kDa MW centrifugal filter (Amicon/Millipore) and all samples were assessed for purity by SDS-PAGE.

Crystallization was performed in 48-well plates using the hanging-drop vapour diffusion method and sparse matrix screen Hampton I and II, molecular dimensions Proplex and Pact screens and incubated at 23°C for a period of four days. The SDR protein was concentrated to 9.7 mg/ml, and screened by combining 1.5 μL of protein and 1.5 μL of precipitant solution over 300 μL of reservoir solution. Crystals formed in 0.2 M lithium sulphate monohydrate, 0.1 M TRIS hydrochloride pH 8.5, and 30% (v/v) polyethylene glycol 8,000, cryogenically preserved in 15% glycerol.

### Data collection, structure determination and refinement

X-ray diffraction data was collected at the MX2 beamline at the Australian Synchrotron. Data was indexed and integrated using iMosflm [33] and scaled and reduced in Aimless [34]. Phases were solved by molecular replacement in Phaser [35] and model building and refinement performed in Coot [36] and Phenix [37]. PDBsum was used to investigate the macromolecular structures and interactions.

## Results and discussion

### Expression, purification, and crystallization of an SDR from *Acinetobacter baumannii*

*Acinetobacter baumannii* is a multi-drug resistant and medically important bacterium. The protein investigated in this study is a short chain dehydrogenase (NCBI Reference Sequence WP_057046512.1; Uniprot A0A7U4DHS9; KEEG AB57_2576), and the structure has not been determined. Since the closest known protein structure exhibits <50% sequence identity, we determined the structure to expand the potential number of drug targets for *in silico* drug design and modelling. Crystals diffracting to 2.5 Å (see Table 1 for data collection and refinement statistics) were indexed in P 6422, and phases solved by molecular replacement using a monomer of an SDR enzyme from *Bacillus anthracis* (PDB: 3T4X), whose sequence was 44% similar to the SDR in this study. A final structural model was refined to R-work of 24.9% and an R-free of 26.5%, and has been deposited to the Protein Data Bank and issued the code 8G9M.

**Table 1:** Data collection and refinement statistics.

Parameters	Value
Space group	P 64 2 2
Resolution Range (Å)	29.64–2.50 (2.60–2.50)
Unit cell length (Å)	93.77 93.77 190.99
Total observations	141291 (16330)
Unique observations	17882 (1979)
Multiplicity	7.9 (8.3)
Completeness (%)	99.9 (100)
Mean I/ Sigma (I)	11.3 (2.0)
Rpim	0.042 (0.418)
CC1/2	0.999 (0.896)
**Refinement**	
R-work	0.2492
R-free	0.2652
RMSD bonds (A)	0.002
RMSD angles (*)	0.508
Ramachandran favoured (%)	97.16
Ramachandran allowed (%)	2.84
Ramachandran outliers (%)	0.00

### Structural analysis of *Acinetobacter baumannii* SDR

**Fig 1:**
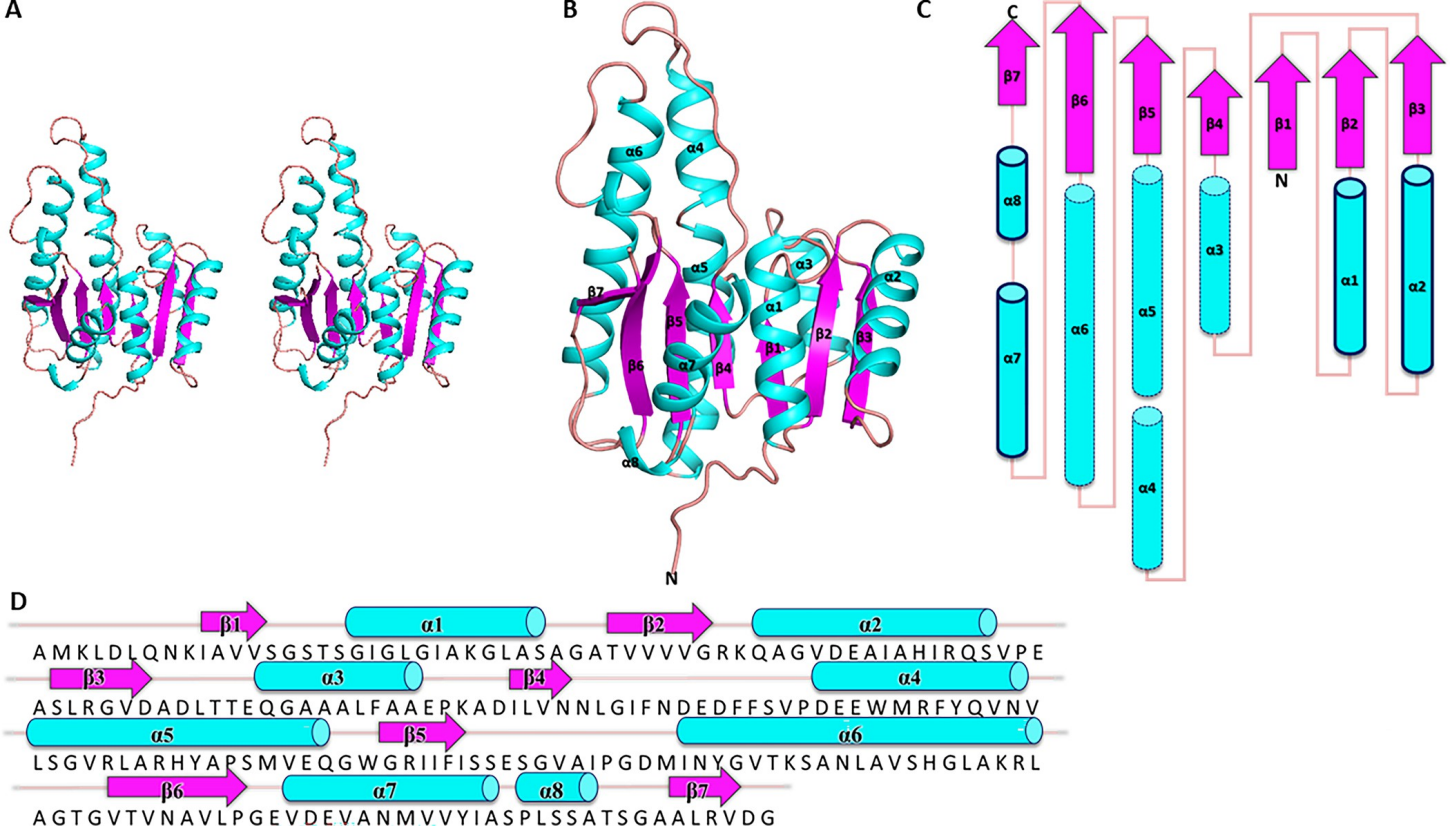
Secondary and tertiary structural elements of the an *Acinetobacter baumannii* SDR. The α-helices are labelled in cyan, β-strands in purple, and loops in light brown. A. Stereo-view of the tertiary structure. B. The tertiary structure of the enzyme shown in cartoon mode, highlighting a central seven-stranded parallel β-sheet, sandwiched between two groups of α helices. C. Topology diagram with colouring matched to A. Helices α1, α2, α7, α8 are on one face and bold, and α3, α4, α5, α6 on the other face with dashed lines. D. Sequence of the SDR with aligned secondary structural elements, and colouring as per A and B.

The enzyme exhibited all the structural features of a typical SDR family member. The protomeric unit was comprised of a central seven-stranded parallel β-sheet, sandwiched between two groups of α helices (α1, α2, α7, α8 on one face, and α3, α4, α5, α6 on the other face), with an overall topology of β1-α1-β2-α2-β3-α3-β4-α4-α5-β5-α6-β6-α7-α8 (Fig 1).

**Fig 2:**
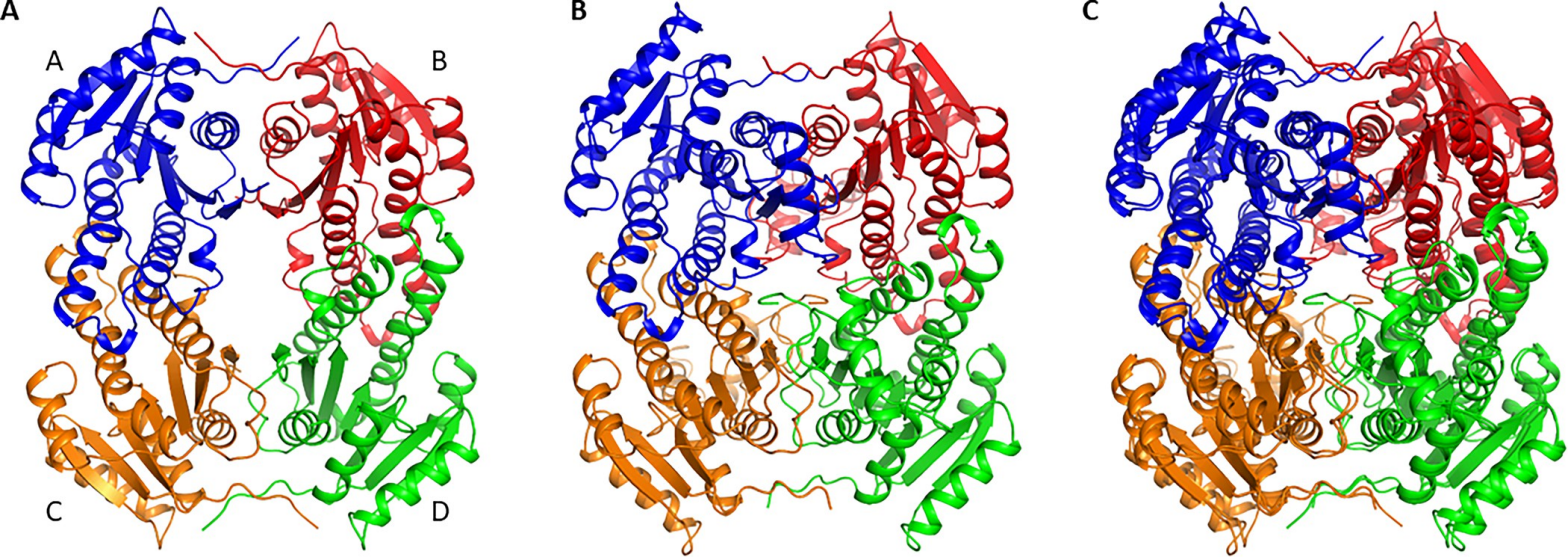
*Acinetobacter baumannii* SDR forms a tetramer. A) Cartoon of the *Acinetobacter baumannii* SDR biological tetramer. Each protomer is labelled A-D and coloured separately B) Structural comparison showing the same tetrameric complex in the SDR family oxidoreductase from *Bacillus anthracis* with 45% sequence similarity and 1.1 Å rmsd. C) Overlay of the two SDR enzymes.

While the asymmetric unit contained a single protomer, analysis of the structure in Proteins, Interfaces, Structures and Assemblies (PISA) [38,39] revealed a tetramer, that was consistent with other SDR enzymes. This tetramer was mediated by two types of interfaces, labelled as A/B, A/C (Fig 2 and S1 Table). The A/B interface was mediated by 8 hydrogen bonds and 112 non-bonded contacts, 22 interfacing residues, and buried 1,243Å^2^ of surface area (S1 Table). Key interactions include Lys^2^ bonding with Asp^4^ and Asn^235^; Glu^232^ bonding with Ser^246^; and Thr^248^ bonding with Arg^254^ (S1 Table). The A/C interface was mediated by 8 hydrogen bonds, 6 salt bridges, 167 non-bonded contacts, 27 interacting residues, and 1,580Å^2^ of buried surface area. Key interactions include Asp^97^ bonding Arg1^71^; and Asp^103^ bonding with Arg^122^ and Arg^119^.

**Fig 3:**
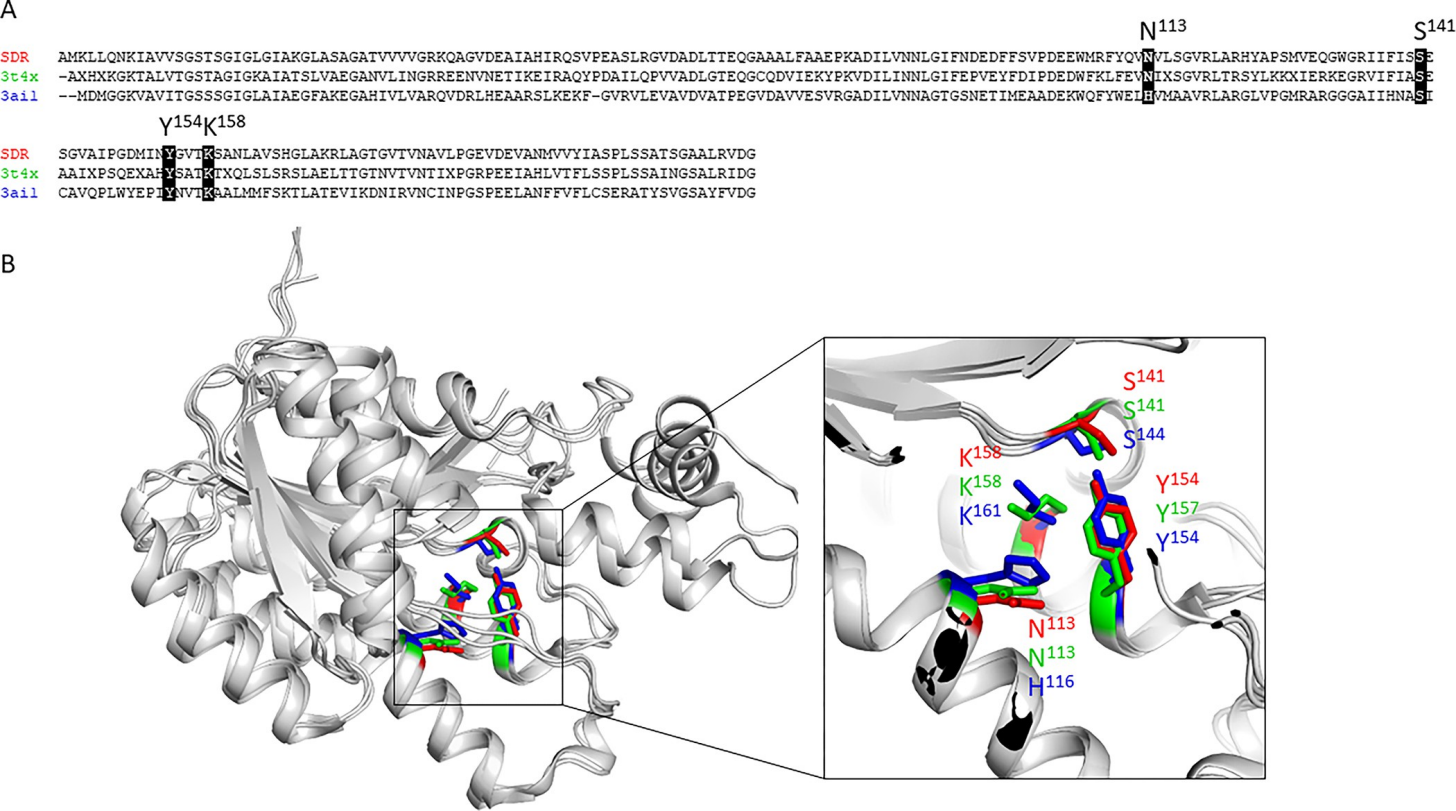
Alignment of the *Acinetobacter baumannii* SDR with the two structurally related SDR enzymes from the DALI search. A) Sequence alignment based on the *Acinetobacter baumannii* SDR from this study (red), an SDR family oxidoreductase from *Bacillus anthracis* with 45% sequence similarity (1.1 Å rmsd) (PDB code: 3T4X; unpublished; blue), and the oxidoreductase from *Gluconobacter frateurii* 3AI1 (1.5 Å rmsd; sequence identity 35%). B) Alignment of active site residues with colouring as per panel A.

Since the structure of this *A*. *baumannii* SDR has not been studied previously, we used BLAST and DALI to identify similar proteins with sequence and structural similarity respectively. In terms of sequence identity, the closest homologue was an SDR family oxidoreductase from *Acinetobacter pittii*, with 82% sequence identity, followed by *Acinetobacter nosocomialis* and *Acinetobacter oleivorans*, both sharing 81% sequence identity. The most closely related structural protein that has been solved to date is that of an SDR family oxidoreductase from *Bacillus anthracis* with 44% sequence similarity (1.1 Å rmsd) (Fig 3) (PDB code: 3T4X; unpublished, 2.80Å), and the L-sorbose oxidoreductase complexed with NADPH and L-Sorbose from *Gluconobacter frateurii* with PDB codes of 3AI1, 3AI2 and 3AI3 with resolutions of 2.38Å, 1.90Å and 1.80Å, respectively, (1.5 Å rmsd; sequence identity 35%), all of which formed the same tetrameric complex [40]. Whilst many SDRs are tetrameric and exhibit the same structural state as reported in this study, the biological importance of tetramerization is not fully understood. Indeed, that many SDRs exist as dimers, implies that tetramerization is not crucial for enzymatic function. For example, FabG enzymes from *Vibrio cholerae*, *Staphylococcus aureus* and *Mycobacterium tuberculosis* function as dimer [41–43], while the homologous enzyme from *E*. *coli* and *P*. *falciparum* functions as a tetramer [44,45]. Significantly, these interfaces have been targets for drug design [46].

The high-resolution structure of the SDR from Acinetobacter baumannii described here may provide valuable insights for drug design, and echo the success seen with other SDRs targeted in pharmaceutical research. The detailed structural information can enable precise target identification and validation, a critical step as seen in the development of drugs targeting similar enzymes. With the clear delineation of active sites and binding pockets, rational drug design based on structure-based techniques such as molecular docking can predict how potential inhibitors might interact with the enzyme, a strategy that has proven effective in designing inhibitors for other SDRs [47]. The high resolution of the *A*. *baumannii* SDR structure may aid in designing drugs with high selectivity and specificity, mirroring the approach taken for other bacterial and human SDRs, where specificity is crucial to minimize off-target effects. This aspect is particularly relevant, as the study of other SDRs has shown how subtle structural differences can be exploited to develop highly selective inhibitors [48,49]. Moreover, the structure may also prove useful for identification of putative substrates through molecular docking [50].

### Conclusion

In this study, we have elucidated the structure of a specific Short-chain Dehydrogenase/Reductase (SDR) from *A*. *baumannii*. Our findings reveal that this SDR retains classical structural features characteristic of SDRs, particularly in its active site residues. A comparative structural analysis with other protein structures in the Protein Data Bank demonstrates this enzyme’s striking similarity to an L-sorbose SDR. This finding highlights the imperative for more detailed research to precisely determine the enzyme’s substrate and to explore its role in the metabolic pathways of *A*. *baumannii*. This exploration is crucial, not only for understanding the enzyme’s function but also for evaluating its viability as a potential target in drug development. The structural insights gained from our study provide a strong platform for assessing the enzyme’s relevance, particularly in the context of drug development. Should further research establish a central role for this SDR in *A*. *baumannii’s* survival or antibiotic resistance mechanisms, our study provides a critical foundation for the design of new drugs using computational methods. These advancements hold significant potential for addressing the growing challenge of antibiotic resistance in *A*. *baumannii* infections.

## Supporting information

S1 Table Bonds within the A/B and A/C interface. A list of hydrogen bonded and non-hydrogen bonded contacts between the protein-protein interfaces.
(DOCX)
