# Supplementary material for: Deciphering the structure of a multi-drug resistant Acinetobacter baumannii short-chain dehydrogenase reductase
Source: PLoS One. 2024 Feb 23;19(2):e0297751. doi: 10.1371/journal.pone.0297751 (PMC10889901; doi:10.1371/journal.pone.0297751)
Supplement: S1 Table — A list of hydrogen bonded and non-hydrogen bonded contacts between the protein-protein interfaces. (DOCX) [file pone.0297751.s001.docx]

Supplementary Table 1. Bonds within the A/B and A/C interface. A list of hydrogen bonded and non-hydrogen bonded contacts between the protein-protein interfaces.

A/B interface Hydrogen Bonded Contacts

Atom Atom Res Res Atom Atom Res Res

no. name name no. Chain no. name name no. Chain Distance

1. 14 O LYS 2 A <--> 1567 N ASP 4 B 2.50

2. 19 NZ LYS 2 A <--> 2927 OD1 ASN 235 B 2.97

3. 28 N ASP 4 A <--> 1553 O LYS 2 B 2.50

4. 1368 OE2 GLU 232 A <--> 3007 OG SER 246 B 2.17

5. 1388 OD1 ASN 235 A <--> 1558 NZ LYS 2 B 2.97

6. 1468 OG SER 246 A <--> 2907 OE2 GLU 232 B 2.17

7. 1480 OG1 THR 248 A <--> 3051 O ARG 254 B 2.82

8. 1512 O ARG 254 A <--> 3019 OG1 THR 248 B 2.82

A/B interface Non-bonded contacts

Atom Atom Res Res Atom Atom Res Res

no. name name no. Chain no. name name no. Chain Distance

1. 2 CA ALA 0 A <--> 1734 O SER 29 B 3.86

2. 3 C ALA 0 A <--> 1734 O SER 29 B 3.29

3. 4 O ALA 0 A <--> 1734 O SER 29 B 3.61

4. 4 O ALA 0 A <--> 1739 C ALA 30 B 3.37

5. 4 O ALA 0 A <--> 1740 O ALA 30 B 3.37

6. 4 O ALA 0 A <--> 1742 N GLY 31 B 3.30

7. 4 O ALA 0 A <--> 1743 CA GLY 31 B 3.13

8. 5 CB ALA 0 A <--> 1734 O SER 29 B 3.68

9. 6 N MET 1 A <--> 1734 O SER 29 B 3.21

10. 7 CA MET 1 A <--> 1734 O SER 29 B 3.51

11. 8 C MET 1 A <--> 1734 O SER 29 B 3.55

12. 9 O MET 1 A <--> 1734 O SER 29 B 3.49

13. 13 C LYS 2 A <--> 1567 N ASP 4 B 3.67

14. 14 O LYS 2 A <--> 1560 CA LEU 3 B 3.33

15. 14 O LYS 2 A <--> 1561 C LEU 3 B 3.32

16. 14 O LYS 2 A <--> 1567 N ASP 4 B 2.50

17. 14 O LYS 2 A <--> 1568 CA ASP 4 B 3.45

18. 14 O LYS 2 A <--> 1570 CB ASP 4 B 3.54

19. 16 CG LYS 2 A <--> 1736 OG SER 29 B 3.60

20. 17 CD LYS 2 A <--> 1717 O GLY 26 B 3.70

21. 17 CD LYS 2 A <--> 1736 OG SER 29 B 3.14

22. 19 NZ LYS 2 A <--> 2927 OD1 ASN 235 B 2.97

23. 21 CA LEU 3 A <--> 1553 O LYS 2 B 3.33

24. 22 C LEU 3 A <--> 1553 O LYS 2 B 3.32

25. 28 N ASP 4 A <--> 1552 C LYS 2 B 3.67

26. 28 N ASP 4 A <--> 1553 O LYS 2 B 2.50

27. 29 CA ASP 4 A <--> 1553 O LYS 2 B 3.45

28. 31 CB ASP 4 A <--> 1553 O LYS 2 B 3.54

29. 178 O GLY 26 A <--> 1556 CD LYS 2 B 3.70

30. 195 O SER 29 A <--> 1541 CA ALA 0 B 3.86

31. 195 O SER 29 A <--> 1542 C ALA 0 B 3.29

32. 195 O SER 29 A <--> 1543 O ALA 0 B 3.61

33. 195 O SER 29 A <--> 1544 CB ALA 0 B 3.68

34. 195 O SER 29 A <--> 1545 N MET 1 B 3.21

35. 195 O SER 29 A <--> 1546 CA MET 1 B 3.51

36. 195 O SER 29 A <--> 1547 C MET 1 B 3.55

37. 195 O SER 29 A <--> 1548 O MET 1 B 3.49

38. 197 OG SER 29 A <--> 1555 CG LYS 2 B 3.60

39. 197 OG SER 29 A <--> 1556 CD LYS 2 B 3.14

40. 200 C ALA 30 A <--> 1543 O ALA 0 B 3.37

41. 201 O ALA 30 A <--> 1543 O ALA 0 B 3.37

42. 203 N GLY 31 A <--> 1543 O ALA 0 B 3.30

43. 204 CA GLY 31 A <--> 1543 O ALA 0 B 3.13

44. 1364 CB GLU 232 A <--> 3012 CB ALA 247 B 3.73

45. 1365 CG GLU 232 A <--> 3007 OG SER 246 B 3.26

46. 1366 CD GLU 232 A <--> 3007 OG SER 246 B 3.02

47. 1366 CD GLU 232 A <--> 3012 CB ALA 247 B 3.90

48. 1368 OE2 GLU 232 A <--> 3004 C SER 246 B 3.88

49. 1368 OE2 GLU 232 A <--> 3006 CB SER 246 B 3.49

50. 1368 OE2 GLU 232 A <--> 3007 OG SER 246 B 2.17

51. 1383 C ASN 235 A <--> 2961 OH TYR 239 B 3.47

52. 1384 O ASN 235 A <--> 2961 OH TYR 239 B 3.72

53. 1385 CB ASN 235 A <--> 2961 OH TYR 239 B 3.80

54. 1385 CB ASN 235 A <--> 2991 O LEU 244 B 3.71

55. 1388 OD1 ASN 235 A <--> 1558 NZ LYS 2 B 2.97

56. 1387 ND2 ASN 235 A <--> 2989 CA LEU 244 B 3.62

57. 1387 ND2 ASN 235 A <--> 2991 O LEU 244 B 3.39

58. 1387 ND2 ASN 235 A <--> 2992 CB LEU 244 B 3.64

59. 1387 ND2 ASN 235 A <--> 2995 CD2 LEU 244 B 3.53

60. 1389 N MET 236 A <--> 2961 OH TYR 239 B 3.21

61. 1390 CA MET 236 A <--> 2961 OH TYR 239 B 3.26

62. 1393 CB MET 236 A <--> 2961 OH TYR 239 B 3.67

63. 1415 CB TYR 239 A <--> 2956 CD1 TYR 239 B 3.73

64. 1415 CB TYR 239 A <--> 2958 CE1 TYR 239 B 3.76

65. 1416 CG TYR 239 A <--> 2955 CG TYR 239 B 3.88

66. 1416 CG TYR 239 A <--> 2956 CD1 TYR 239 B 3.89

67. 1417 CD1 TYR 239 A <--> 2954 CB TYR 239 B 3.73

68. 1417 CD1 TYR 239 A <--> 2955 CG TYR 239 B 3.89

69. 1419 CE1 TYR 239 A <--> 2954 CB TYR 239 B 3.76

70. 1422 OH TYR 239 A <--> 2922 C ASN 235 B 3.47

71. 1422 OH TYR 239 A <--> 2923 O ASN 235 B 3.72

72. 1422 OH TYR 239 A <--> 2924 CB ASN 235 B 3.80

73. 1422 OH TYR 239 A <--> 2928 N MET 236 B 3.21

74. 1422 OH TYR 239 A <--> 2929 CA MET 236 B 3.26

75. 1422 OH TYR 239 A <--> 2932 CB MET 236 B 3.67

76. 1450 CA LEU 244 A <--> 2926 ND2 ASN 235 B 3.62

77. 1452 O LEU 244 A <--> 2924 CB ASN 235 B 3.71

78. 1452 O LEU 244 A <--> 2926 ND2 ASN 235 B 3.39

79. 1453 CB LEU 244 A <--> 2926 ND2 ASN 235 B 3.64

80. 1456 CD2 LEU 244 A <--> 2926 ND2 ASN 235 B 3.53

81. 1465 C SER 246 A <--> 2907 OE2 GLU 232 B 3.88

82. 1467 CB SER 246 A <--> 2907 OE2 GLU 232 B 3.49

83. 1468 OG SER 246 A <--> 2904 CG GLU 232 B 3.26

84. 1468 OG SER 246 A <--> 2905 CD GLU 232 B 3.02

85. 1468 OG SER 246 A <--> 2907 OE2 GLU 232 B 2.17

86. 1471 C ALA 247 A <--> 3069 O ASP 256 B 3.56

87. 1472 O ALA 247 A <--> 3060 CA VAL 255 B 3.27

88. 1472 O ALA 247 A <--> 3061 C VAL 255 B 3.32

89. 1472 O ALA 247 A <--> 3062 O VAL 255 B 3.77

90. 1472 O ALA 247 A <--> 3066 N ASP 256 B 3.63

91. 1472 O ALA 247 A <--> 3068 C ASP 256 B 3.67

92. 1472 O ALA 247 A <--> 3069 O ASP 256 B 2.69

93. 1473 CB ALA 247 A <--> 2903 CB GLU 232 B 3.73

94. 1473 CB ALA 247 A <--> 2905 CD GLU 232 B 3.90

95. 1478 CB THR 248 A <--> 3051 O ARG 254 B 3.76

96. 1480 OG1 THR 248 A <--> 3051 O ARG 254 B 2.82

97. 1480 OG1 THR 248 A <--> 3064 CG1 VAL 255 B 3.54

98. 1479 CG2 THR 248 A <--> 3051 O ARG 254 B 3.80

99. 1490 O GLY 250 A <--> 3057 NH1 ARG 254 B 3.40

100. 1508 CD2 LEU 253 A <--> 3047 CD2 LEU 253 B 3.73

101. 1512 O ARG 254 A <--> 3017 CB THR 248 B 3.76

102. 1512 O ARG 254 A <--> 3019 OG1 THR 248 B 2.82

103. 1512 O ARG 254 A <--> 3018 CG2 THR 248 B 3.80

104. 1518 NH1 ARG 254 A <--> 3029 O GLY 250 B 3.40

105. 1521 CA VAL 255 A <--> 3011 O ALA 247 B 3.27

106. 1522 C VAL 255 A <--> 3011 O ALA 247 B 3.32

107. 1523 O VAL 255 A <--> 3011 O ALA 247 B 3.77

108. 1525 CG1 VAL 255 A <--> 3019 OG1 THR 248 B 3.54

109. 1527 N ASP 256 A <--> 3011 O ALA 247 B 3.63

110. 1529 C ASP 256 A <--> 3011 O ALA 247 B 3.67

111. 1530 O ASP 256 A <--> 3010 C ALA 247 B 3.56

112. 1530 O ASP 256 A <--> 3011 O ALA 247 B 2.69

A/C interface Hydrogen Bonded Contacts

Atom Atom Res Res Atom Atom Res Res

no. name name no. Chain no. name name no. Chain Distance

1. 673 OD1 ASP 97 A <--> 4322 NE ARG 171 C 2.92

2. 674 OD2 ASP 97 A <--> 4325 NH2 ARG 171 C 3.34

3. 723 OD1 ASP 103 A <--> 3967 NH2 ARG 122 C 2.28

4. 724 OD2 ASP 103 A <--> 3942 NH1 ARG 119 C 2.49

5. 864 NH1 ARG 119 A <--> 3802 OD2 ASP 103 C 2.49

6. 889 NH2 ARG 122 A <--> 3801 OD1 ASP 103 C 2.28

7. 1244 NE ARG 171 A <--> 3751 OD1 ASP 97 C 2.92

8. 1247 NH2 ARG 171 A <--> 3752 OD2 ASP 97 C 3.34

A/C interface Non-Bonded Contacts

Atom Atom Res Res Atom Atom Res Res

no. name name no. Chain no. name name no. Chain Distance

1. 672 CG ASP 97 A <--> 4322 NE ARG 171 C 3.76

2. 672 CG ASP 97 A <--> 4325 NH2 ARG 171 C 3.77

3. 673 OD1 ASP 97 A <--> 4321 CD ARG 171 C 3.88

4. 673 OD1 ASP 97 A <--> 4322 NE ARG 171 C 2.92

5. 673 OD1 ASP 97 A <--> 4323 CZ ARG 171 C 3.52

6. 673 OD1 ASP 97 A <--> 4325 NH2 ARG 171 C 3.40

7. 674 OD2 ASP 97 A <--> 4322 NE ARG 171 C 3.87

8. 674 OD2 ASP 97 A <--> 4325 NH2 ARG 171 C 3.34

9. 682 CD2 PHE 98 A <--> 4299 CD1 LEU 168 C 3.90

10. 683 CE1 PHE 98 A <--> 3931 CG1 VAL 118 C 3.78

11. 684 CE2 PHE 98 A <--> 3958 CA ARG 122 C 3.88

12. 684 CE2 PHE 98 A <--> 4300 CD2 LEU 168 C 3.61

13. 685 CZ PHE 98 A <--> 4300 CD2 LEU 168 C 3.74

14. 692 CD1 PHE 99 A <--> 3960 O ARG 122 C 3.78

15. 693 CD2 PHE 99 A <--> 4325 NH2 ARG 171 C 3.46

16. 694 CE1 PHE 99 A <--> 3994 CB ALA 125 C 3.53

17. 695 CE2 PHE 99 A <--> 4323 CZ ARG 171 C 3.80

18. 695 CE2 PHE 99 A <--> 4325 NH2 ARG 171 C 3.62

19. 696 CZ PHE 99 A <--> 3994 CB ALA 125 C 3.68

20. 706 O VAL 101 A <--> 3966 NH1 ARG 122 C 3.39

21. 721 CB ASP 103 A <--> 3915 CD2 LEU 115 C 3.55

22. 722 CG ASP 103 A <--> 3915 CD2 LEU 115 C 3.84

23. 722 CG ASP 103 A <--> 3942 NH1 ARG 119 C 3.54

24. 722 CG ASP 103 A <--> 3967 NH2 ARG 122 C 3.44

25. 723 OD1 ASP 103 A <--> 3965 CZ ARG 122 C 3.28

26. 723 OD1 ASP 103 A <--> 3966 NH1 ARG 122 C 3.54

27. 723 OD1 ASP 103 A <--> 3967 NH2 ARG 122 C 2.28

28. 724 OD2 ASP 103 A <--> 3938 CG ARG 119 C 3.84

29. 724 OD2 ASP 103 A <--> 3939 CD ARG 119 C 3.75

30. 724 OD2 ASP 103 A <--> 3941 CZ ARG 119 C 3.68

31. 724 OD2 ASP 103 A <--> 3942 NH1 ARG 119 C 2.49

32. 746 O TRP 106 A <--> 3874 CE2 TYR 110 C 3.67

33. 747 CB TRP 106 A <--> 3914 CD1 LEU 115 C 3.63

34. 748 CG TRP 106 A <--> 3914 CD1 LEU 115 C 3.75

35. 753 NE1 TRP 106 A <--> 3931 CG1 VAL 118 C 3.73

36. 753 NE1 TRP 106 A <--> 3966 NH1 ARG 122 C 3.75

37. 751 CE2 TRP 106 A <--> 3931 CG1 VAL 118 C 3.65

38. 752 CE3 TRP 106 A <--> 3876 OH TYR 110 C 3.71

39. 754 CZ2 TRP 106 A <--> 3931 CG1 VAL 118 C 3.79

40. 760 O MET 107 A <--> 3842 CE MET 107 C 3.62

41. 763 SD MET 107 A <--> 3881 CB GLN 111 C 3.69

42. 764 CE MET 107 A <--> 3838 O MET 107 C 3.62

43. 764 CE MET 107 A <--> 3842 CE MET 107 C 3.82

44. 764 CE MET 107 A <--> 3881 CB GLN 111 C 3.75

45. 764 CE MET 107 A <--> 3884 NE2 GLN 111 C 3.89

46. 791 CB TYR 110 A <--> 3872 CD2 TYR 110 C 3.53

47. 791 CB TYR 110 A <--> 3874 CE2 TYR 110 C 3.42

48. 791 CB TYR 110 A <--> 3875 CZ TYR 110 C 3.88

49. 792 CG TYR 110 A <--> 3870 CG TYR 110 C 3.47

50. 792 CG TYR 110 A <--> 3871 CD1 TYR 110 C 3.70

51. 792 CG TYR 110 A <--> 3872 CD2 TYR 110 C 3.46

52. 792 CG TYR 110 A <--> 3874 CE2 TYR 110 C 3.70

53. 793 CD1 TYR 110 A <--> 3870 CG TYR 110 C 3.70

54. 793 CD1 TYR 110 A <--> 3871 CD1 TYR 110 C 3.39

55. 793 CD1 TYR 110 A <--> 3873 CE1 TYR 110 C 3.42

56. 793 CD1 TYR 110 A <--> 3875 CZ TYR 110 C 3.73

57. 794 CD2 TYR 110 A <--> 3869 CB TYR 110 C 3.53

58. 794 CD2 TYR 110 A <--> 3870 CG TYR 110 C 3.46

59. 794 CD2 TYR 110 A <--> 3872 CD2 TYR 110 C 3.68

60. 795 CE1 TYR 110 A <--> 3871 CD1 TYR 110 C 3.42

61. 795 CE1 TYR 110 A <--> 3873 CE1 TYR 110 C 3.75

62. 796 CE2 TYR 110 A <--> 3824 O TRP 106 C 3.67

63. 796 CE2 TYR 110 A <--> 3869 CB TYR 110 C 3.42

64. 796 CE2 TYR 110 A <--> 3870 CG TYR 110 C 3.70

65. 797 CZ TYR 110 A <--> 3869 CB TYR 110 C 3.88

66. 797 CZ TYR 110 A <--> 3871 CD1 TYR 110 C 3.73

67. 798 OH TYR 110 A <--> 3830 CE3 TRP 106 C 3.71

68. 798 OH TYR 110 A <--> 4220 CG1 VAL 156 C 3.56

69. 803 CB GLN 111 A <--> 3841 SD MET 107 C 3.69

70. 803 CB GLN 111 A <--> 3842 CE MET 107 C 3.75

71. 806 NE2 GLN 111 A <--> 3842 CE MET 107 C 3.89

72. 836 CD1 LEU 115 A <--> 3825 CB TRP 106 C 3.63

73. 836 CD1 LEU 115 A <--> 3826 CG TRP 106 C 3.75

74. 837 CD2 LEU 115 A <--> 3799 CB ASP 103 C 3.55

75. 837 CD2 LEU 115 A <--> 3800 CG ASP 103 C 3.84

76. 853 CG1 VAL 118 A <--> 3761 CE1 PHE 98 C 3.78

77. 853 CG1 VAL 118 A <--> 3831 NE1 TRP 106 C 3.73

78. 853 CG1 VAL 118 A <--> 3829 CE2 TRP 106 C 3.65

79. 853 CG1 VAL 118 A <--> 3832 CZ2 TRP 106 C 3.79

80. 860 CG ARG 119 A <--> 3802 OD2 ASP 103 C 3.84

81. 861 CD ARG 119 A <--> 3802 OD2 ASP 103 C 3.75

82. 863 CZ ARG 119 A <--> 3802 OD2 ASP 103 C 3.68

83. 864 NH1 ARG 119 A <--> 3800 CG ASP 103 C 3.54

84. 864 NH1 ARG 119 A <--> 3802 OD2 ASP 103 C 2.49

85. 880 CA ARG 122 A <--> 3762 CE2 PHE 98 C 3.88

86. 882 O ARG 122 A <--> 3770 CD1 PHE 99 C 3.78

87. 887 CZ ARG 122 A <--> 3801 OD1 ASP 103 C 3.28

88. 888 NH1 ARG 122 A <--> 3784 O VAL 101 C 3.39

89. 888 NH1 ARG 122 A <--> 3801 OD1 ASP 103 C 3.54

90. 888 NH1 ARG 122 A <--> 3831 NE1 TRP 106 C 3.75

91. 889 NH2 ARG 122 A <--> 3800 CG ASP 103 C 3.44

92. 889 NH2 ARG 122 A <--> 3801 OD1 ASP 103 C 2.28

93. 916 CB ALA 125 A <--> 3772 CE1 PHE 99 C 3.53

94. 916 CB ALA 125 A <--> 3774 CZ PHE 99 C 3.68

95. 1073 O ILE 147 A <--> 4312 CD LYS 170 C 3.78

96. 1073 O ILE 147 A <--> 4313 CE LYS 170 C 3.08

97. 1073 O ILE 147 A <--> 4314 NZ LYS 170 C 3.79

98. 1074 CB ILE 147 A <--> 4264 O ALA 163 C 3.73

99. 1074 CB ILE 147 A <--> 4289 N GLY 167 C 3.89

100. 1074 CB ILE 147 A <--> 4290 CA GLY 167 C 3.89

101. 1075 CG1 ILE 147 A <--> 4262 CA ALA 163 C 3.58

102. 1075 CG1 ILE 147 A <--> 4263 C ALA 163 C 3.90

103. 1075 CG1 ILE 147 A <--> 4264 O ALA 163 C 3.43

104. 1075 CG1 ILE 147 A <--> 4265 CB ALA 163 C 3.49

105. 1076 CG2 ILE 147 A <--> 4264 O ALA 163 C 3.82

106. 1076 CG2 ILE 147 A <--> 4290 CA GLY 167 C 3.74

107. 1077 CD1 ILE 147 A <--> 4265 CB ALA 163 C 3.79

108. 1081 O PRO 148 A <--> 4310 CB LYS 170 C 3.55

109. 1081 O PRO 148 A <--> 4312 CD LYS 170 C 3.22

110. 1081 O PRO 148 A <--> 4313 CE LYS 170 C 3.85

111. 1086 CA GLY 149 A <--> 4292 O GLY 167 C 3.41

112. 1088 O GLY 149 A <--> 4319 CB ARG 171 C 3.41

113. 1088 O GLY 149 A <--> 4321 CD ARG 171 C 3.85

114. 1112 CD1 ILE 152 A <--> 4291 C GLY 167 C 3.56

115. 1112 CD1 ILE 152 A <--> 4292 O GLY 167 C 3.56

116. 1112 CD1 ILE 152 A <--> 4293 N LEU 168 C 3.67

117. 1112 CD1 ILE 152 A <--> 4294 CA LEU 168 C 3.84

118. 1138 CA VAL 156 A <--> 4265 CB ALA 163 C 3.83

119. 1142 CG1 VAL 156 A <--> 3876 OH TYR 110 C 3.56

120. 1142 CG1 VAL 156 A <--> 4244 CB ALA 160 C 3.89

121. 1143 CG2 VAL 156 A <--> 4265 CB ALA 163 C 3.73

122. 1143 CG2 VAL 156 A <--> 4266 N VAL 164 C 3.78

123. 1143 CG2 VAL 156 A <--> 4272 CG2 VAL 164 C 3.56

124. 1161 OG SER 159 A <--> 4265 CB ALA 163 C 3.17

125. 1166 CB ALA 160 A <--> 4220 CG1 VAL 156 C 3.89

126. 1184 CA ALA 163 A <--> 4153 CG1 ILE 147 C 3.58

127. 1185 C ALA 163 A <--> 4153 CG1 ILE 147 C 3.90

128. 1186 O ALA 163 A <--> 4152 CB ILE 147 C 3.73

129. 1186 O ALA 163 A <--> 4153 CG1 ILE 147 C 3.43

130. 1186 O ALA 163 A <--> 4154 CG2 ILE 147 C 3.82

131. 1187 CB ALA 163 A <--> 4153 CG1 ILE 147 C 3.49

132. 1187 CB ALA 163 A <--> 4155 CD1 ILE 147 C 3.79

133. 1187 CB ALA 163 A <--> 4216 CA VAL 156 C 3.83

134. 1187 CB ALA 163 A <--> 4221 CG2 VAL 156 C 3.73

135. 1187 CB ALA 163 A <--> 4239 OG SER 159 C 3.17

136. 1188 N VAL 164 A <--> 4221 CG2 VAL 156 C 3.78

137. 1194 CG2 VAL 164 A <--> 4221 CG2 VAL 156 C 3.56

138. 1211 N GLY 167 A <--> 4152 CB ILE 147 C 3.89

139. 1212 CA GLY 167 A <--> 4152 CB ILE 147 C 3.89

140. 1212 CA GLY 167 A <--> 4154 CG2 ILE 147 C 3.74

141. 1213 C GLY 167 A <--> 4190 CD1 ILE 152 C 3.56

142. 1214 O GLY 167 A <--> 4164 CA GLY 149 C 3.41

143. 1214 O GLY 167 A <--> 4190 CD1 ILE 152 C 3.56

144. 1215 N LEU 168 A <--> 4190 CD1 ILE 152 C 3.67

145. 1216 CA LEU 168 A <--> 4190 CD1 ILE 152 C 3.84

146. 1221 CD1 LEU 168 A <--> 3760 CD2 PHE 98 C 3.90

147. 1222 CD2 LEU 168 A <--> 3762 CE2 PHE 98 C 3.61

148. 1222 CD2 LEU 168 A <--> 3763 CZ PHE 98 C 3.74

149. 1232 CB LYS 170 A <--> 4159 O PRO 148 C 3.55

150. 1234 CD LYS 170 A <--> 4151 O ILE 147 C 3.78

151. 1234 CD LYS 170 A <--> 4159 O PRO 148 C 3.22

152. 1235 CE LYS 170 A <--> 4151 O ILE 147 C 3.08

153. 1235 CE LYS 170 A <--> 4159 O PRO 148 C 3.85

154. 1236 NZ LYS 170 A <--> 4151 O ILE 147 C 3.79

155. 1241 CB ARG 171 A <--> 4166 O GLY 149 C 3.41

156. 1243 CD ARG 171 A <--> 3751 OD1 ASP 97 C 3.88

157. 1243 CD ARG 171 A <--> 4166 O GLY 149 C 3.85

158. 1244 NE ARG 171 A <--> 3750 CG ASP 97 C 3.76

159. 1244 NE ARG 171 A <--> 3751 OD1 ASP 97 C 2.92

160. 1244 NE ARG 171 A <--> 3752 OD2 ASP 97 C 3.87

161. 1245 CZ ARG 171 A <--> 3751 OD1 ASP 97 C 3.52

162. 1245 CZ ARG 171 A <--> 3773 CE2 PHE 99 C 3.80

163. 1247 NH2 ARG 171 A <--> 3750 CG ASP 97 C 3.77

164. 1247 NH2 ARG 171 A <--> 3751 OD1 ASP 97 C 3.40

165. 1247 NH2 ARG 171 A <--> 3752 OD2 ASP 97 C 3.34

166. 1247 NH2 ARG 171 A <--> 3771 CD2 PHE 99 C 3.46

167. 1247 NH2 ARG 171 A <--> 3773 CE2 PHE 99 C 3.62

A/C Salt Bridges

Atom Atom Res Res Atom Atom Res Res

no. name name no. Chain no. name name no. Chain Distance

1. 673 OD1 ASP 97 A <--> 4322 NE ARG 171 C 2.92

2. 724 OD2 ASP 103 A <--> 3942 NH1 ARG 119 C 2.49

3. 723 OD1 ASP 103 A <--> 3967 NH2 ARG 122 C 2.28

4. 864 NH1 ARG 119 A <--> 3802 OD2 ASP 103 C 2.49

5. 889 NH2 ARG 122 A <--> 3801 OD1 ASP 103 C 2.28

6. 1244 NE ARG 171 A <--> 3751 OD1 ASP 97 C 2.92
